# Mid-term Clinical and Radiological Results of Oxford Phase 3 Medial Unicompartmental Knee Arthroplasty

**DOI:** 10.7759/cureus.5674

**Published:** 2019-09-16

**Authors:** Yusuf Erdem, Cagri Neyisci, Cemil Yıldız

**Affiliations:** 1 Orthopaedics, Gulhane Training and Research Hospital, Ankara, TUR; 2 Orthopaedics and Traumatology, Gulhane Training and Research Hospital, Ankara, TUR

**Keywords:** unicompartmental knee arthroplasty, medial compartment, osteoarthritis

## Abstract

Introduction

The popularity of unicompartmental knee arthroplasty (UKA) for the treatment of isolated compartment osteoarthritis of the knee has risen over the past two decades. Currently, UKA covers a considerable amount of all knee arthroplasties worldwide. The aim of this study was to present the clinical and radiological outcomes of UKA in patients with medial compartment osteoarthritis.

Methods

Between January 2010 and January 2014, mobile-bearing UKA was applied to 44 knees of 37 (three men, 34 women) patients with isolated medial compartment osteoarthritis. The mean age, body mass index (BMI), and follow-up were 54 ± 6.1, 26.3± 2.2, and 48 ± 9.4, respectively. Oxford Knee Score (OKS), Knee Society Score (KSS), visual analog scale (VAS), and range of motion (ROM) were used for clinical assessment, and the Oxford Radiological Evaluation Criteria were used for radiological assessment.

Results

Compared to preoperative values, knee flexion increased from 116° to 123° (p<0.001). Statistically significant increases in OKS and KSS and decrease in VAS was obtained postoperatively (p<0.001). All of the components were aligned within the acceptable ranges radiologically. One bearing dislocation was revised and one conversion to TKA was performed during the 5.9-year follow-up. No major complications occurred, including infection, deep vein thrombosis, pulmonary emboli, and neurovascular injury.

Conclusion

The mid-term clinical and radiological outcomes of UKA were excellent in this study, and our results demonstrate that Oxford mobile-bearing UKA for the proper indication is effective, with considerable success in the treatment of medial compartmental knee osteoarthritis, regardless of age.

## Introduction

Since the first design was introduced by McKeever in 1957 [1], the unicompartmental knee arthroplasty (UKA) technique has been developed with more anatomical implants and the minimally invasive approach over the years. Marmor reported 10% revision rates in the two-year follow-up and 65% survivorship with the first design implants in UKA at a mean follow-up of 10 years [2-3] while Goodfellow’s early results with Oxford Phase II was reported by Murray as more than 90% survivorship at 10 years [4]. İncreased success has been achieved by understanding isolated anteromedial arthritis, which is identified as bone cartilage loss in the anterior and mid-portion of the medial compartment in association with intact ligamentous structures and normal lateral compartment cartilage [5-6]. In the late 1990s, more anatomical implants were manufactured and superior outcomes were yielded with these anatomical designs and minimally invasive surgical techniques. In a review, Khanna et al. reported a 93% survival rate in 15 years [7], and in other studies with Oxford implant UKA, survivorship has demonstrated greater than 90% for more than a 15-year follow-up [8-9]. These scientific publications, even with good long-term survival rates by designer surgeons, induced the resurgence in UKA’s popularity over osteotomies and total knee arthroplasties (TKA). On the other hand, polyethylene wear as a failure in fixed-bearing implants has increased the use of mobile-bearing implants in the past two decades [10-12]. Moreover, high success rates, regardless of age and body mass index, have prompted surgeons to utilize mobile bearing implants more than before.

In this study, we retrospectively reviewed the mid-term clinical and radiologic results of minimally invasive Oxford medial UKA performed in the Turkish population, regardless of age. The purpose of this study was to assess the midterm results and survival rates and complications of 44 knees with medial compartment osteoarthritis treated consecutively with Oxford cemented mobile UKA implants.

## Materials and methods

Written, informed consent was obtained from each patient. The study protocol was approved by the Institutional Review Board. The study was conducted in accordance with the principles of the Declaration of Helsinki. Between January 2010 and January 2014, 44 knees of 37 patients underwent medial UKA for the treatment of osteoarthritis. Of the 37 patients (44 knees), seven underwent bilateral UKA and 30 underwent unilateral UKA. All surgeries were performed by the same surgeon using the Oxford Phase III (Biomet, Warsaw, IN, USA) prosthesis.

Clinical and radiographic indications for surgery were refractory pain on one finger test despite conservative treatments in the medial aspect of the knee, varus deformity lower than 15°, flexion contracture lower than 15°, and Outerbridge Grade II medial compartment osteoarthritis, respectively. Exclusion criteria included pain in other compartments rather than the medial aspect of the knee, grade III-IV Outerbridge osteoarthritis, and a history of surgery for osteoarthritis and previous fractures around the affected knee. Age and weight were not considered contraindications for surgery.

Clinical assessment was performed preoperatively, six months after surgery and one year after surgery using the knee range of motion (ROM), visual analog scale (VAS), Oxford Knee Score (OKS) [13], and Knee Society Score (KSS) [14]. For the radiological assessment, Oxford radiological evaluation criteria, including implant positioning, tibiofemoral angle, and slope, were used. The tibiofemoral angle was measured on the weight-bearing long leg radiographs, and component loosening or osteoarthritis in the other compartments was investigated on anteroposterior and lateral radiographs of the knee and patella.

Surgical technique

**Figure 1:**
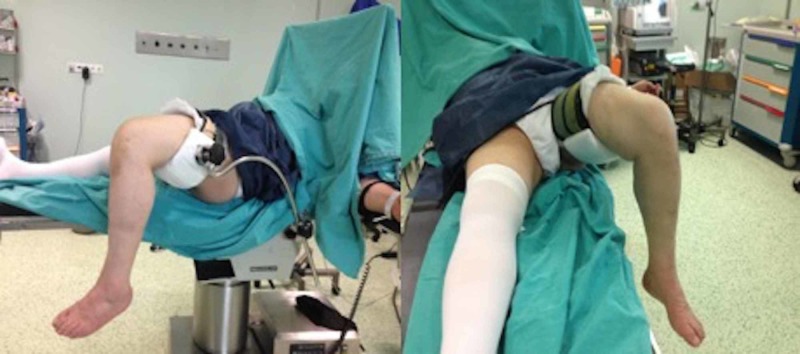
Extremity positioning with leg holder allowing at least 120° flexion

**Figure 2:**
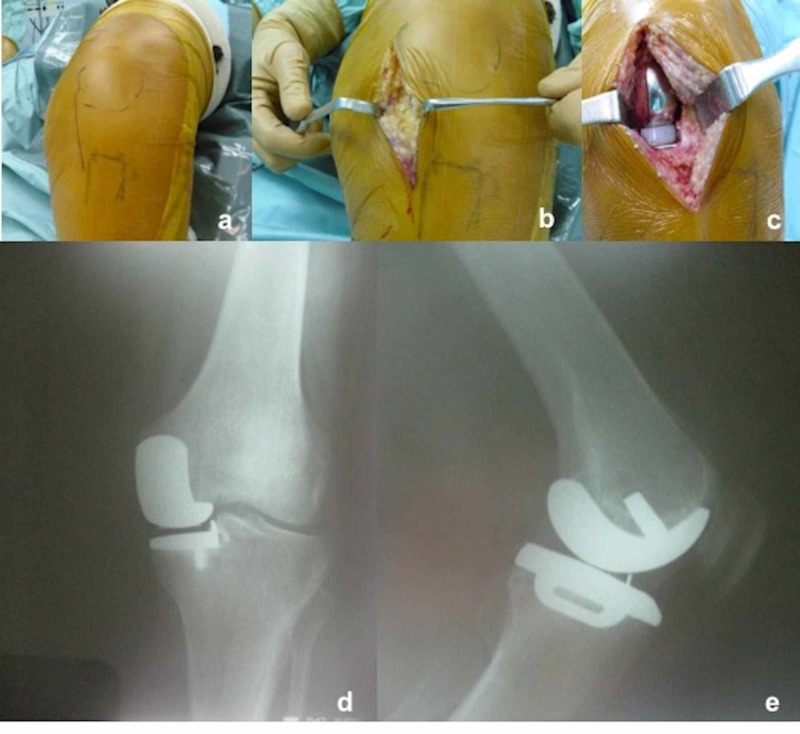
a) Incision marked after draping, b) medial parapatellar incision and arthrotomy, c) placement of the implants, and d,e) postoperative anteroposterior/lateral radiographs of the knee

Patients were placed in the supine position after combined (spinal+epidural) anesthesia in the operating room. The thigh was held in a special leg holder to allow a minimum of 120 degrees knee flexion during the procedure (Figure 1). After the medial parapatellar incision, arthrotomy was performed. Appropriate bone cuts were done and implants were placed (Figure 2). UKA was performed bilaterally in seven patients (14 knees) simultaneously and unilaterally in 30 patients (30 knees). Jones bandage, Ranawat cocktail, and tranexamic acid were applied on all knees in the operating room.

All patients had the same physiotherapy, starting with continuous passive motion (CPM) on the same day after the operation and mobilized with crutches the day after the surgery.

Statistical analysis

Statistical analysis was performed using the IBM SPSS for Mac version 22.0 software (IBM Corp., Armonk, NY, USA). Descriptive data were expressed in mean ± standard deviation (SD), number, and frequency. The t-test of comparison of means for paired data was used to compare the pre- and postoperative ROM, OKS, and KSS scores. A p-value of <0.05 was considered statistically significant.

## Results

Of the patients, there were 34 (92%) females and three (8%) males, with a mean age of 54 ± 6.1 years. The mean follow-up was 70 ± 9.4 months and the mean BMI was 26.3 ± 2.2. The mean operation time was 90 ± 22 minutes. The demographic and clinical characteristics of patients are shown in Table 1. The mean preoperative ROM was 116° ± 5.3° while the mean postoperative ROM was 123° ± 6.4°, and full knee extension was recorded pre- and postoperatively. The mean VAS was decreased from 7.96 ± 1.02 to 2.29 ± 0.98. The mean KSS and OKS were increased from 54.6 ± 7.5 to 90.1 ± 6 and 24.1 ± 3.2 to 52.8 ± 4.6, respectively (Table 2).

**Table 1:** Patients' demographic data

n	37 patients (44 knees)
Age	54 ± 6.1 years
Side	7 bilaterally; 30 unilaterally
Follow-up	70 ± 9.4 months
Gender	34 female (%92), 3 male (%8)
BMI	26.3 ± 2.2
Bearing type	44 mobile
Mean operation time	90 ± 22 minutes

**Table 2:** Postoperative radiological scores

Patient/Sex	Side	Femur Varus/Valgus	Flexion/ Extension		Tibia Varus/Valgus	Slope	Medial Fit
1 (F)	Bilateral	3°/0° varus	0°/0°		0°/0°	87°/88°	Central
2 (F)	Bilateral	1°/0° varus	0°/0°		0°/0°	86°/86°	Central
3 (F)	R	2° varus	0°		0°	88°	Central
4 (F)	L	1° varus	3° flex.		2° varus	87°	Central
5 (F)	L	5° varus	4° flex.		0°	86°	Central
6 (F)	Bilateral	3°/4° varus	1° ext./0°		0°/0°	83°/82°	Central
7 (F)	R	2° valgus	3° ext.		0°	82°	Central
8 (F)	L	0°	0°		0°	85°	Central
9 (F)	L	5° varus	0°		0°	86°	2 mm.
10 (F)	L	4° valgus	3° ext.		1° varus	87°	Central
11 (F)	R	2° varus	0°		0°	88°	Central
12 (F)	Bilateral	6°/4° varus	0°		2°/3° varus	83°/80°	Central
13 (F)	Bilateral	2/2° valgus	0°		0°/0°	84°/80°	Central
14 (M)	R	3° valgus	3° ext.		3° varus	84°	Central
15 (F)	R	0°	0°		0°	83°	1 mm.
16 (F)	R	3° valgus	0°		0°	85°	Central
17 (F)	L	2° varus	0°		0°	86°	Central
18 (F)	L	0°	0°		0°	85°	Central
19 (F)	R	3° valgus	0°		2° varus	86°	Central
20 (F)	R	1° valgus	0°		0°	87°	Central
21 (F)	R	4° valgus	1° flex.		3° valgus	88°	Central
22 (F)	R	0°	0°		0°	81°	Central
23 (F)	R	3° valgus	4° flex.		1° valgus	80°	Central
24 (F)	R	3° varus	2° ext.		1° valgus	84°	Central
25 (F)	L	2° varus	3° ext.		3° valgus	86°	Central
26 (F)	L	3° valgus	2° flex.		0°	84°	1 mm.
27 (F)	L	0°	0°		0°	83°	Central
28 (F)	L	2° varus	0°		0°	81°	Central
29 (M)	R	1° valgus	2° ext.		1° varus	80°	Central
30 (F)	L	2° varus	3° ext.		2° varus	79°	Central
31 (F)	Bilateral	0°/0°	0°/0°		0°/0°	82°/85°	Central
32 (M)	L	2° valgus	4° ext.		0°	83°	Central
33 (F)	Bilateral	0°/0°	0°/0°		0°/0°	82°/84°	Central
34 (F)	R	1° valgus	2° ext.		0°	82°	Central
35 (F)	R	1° varus	2° ext.		1° varus	83°	Central
36 (F)	R	2° varus	3° flex.		0°	84°	1 mm.
37 (F)	R	0°	0°		0°	85°	Central
Preoperative mean femorotibial angle, Postoperative mean femorotibial angle				2.3° ± 0.3° varus, 2.9° ± 0.4° valgus			
Mean tibial slope				84.5° ± 0.2°			

Regarding the radiological assessment, no femoral or tibial component showed radiological loosening. The mean femorotibial angle measured on the weight-bearing radiograph was 2.3° ± 0.3° varus preoperatively and 2.9° ± 0.4° of valgus at the final follow-up. There was no radiographic evidence of ≥2 mm pathological radiolucency around the femoral and tibial components or osteolysis. The mean tibial slope was 84.5° ± 0.2°. All of the tibial components, except an overflow of 1 mm in three and 2 mm in one, showed full congruency with the medial plane (Table 3).

**Table 3:** Pre and postoperative results of pain and function scores ROM: range of motion; VAS: visual analog scale;

Parameter	Preoperative	Postoperative	p*
Mean ROM	116° ± 5,3°	123° ± 6,4°	< 0.05
Mean VAS	7.96 ± 1.02	2.29 ± 0.98	< 0.05
Mean Knee Society Scores	54.6 ± 7.5	90.1 ± 6	< 0.05
Mean Oxford Knee Scores	24.1 ± 3.2	52.8 ± 4.6	< 0.05

**Figure 3:**
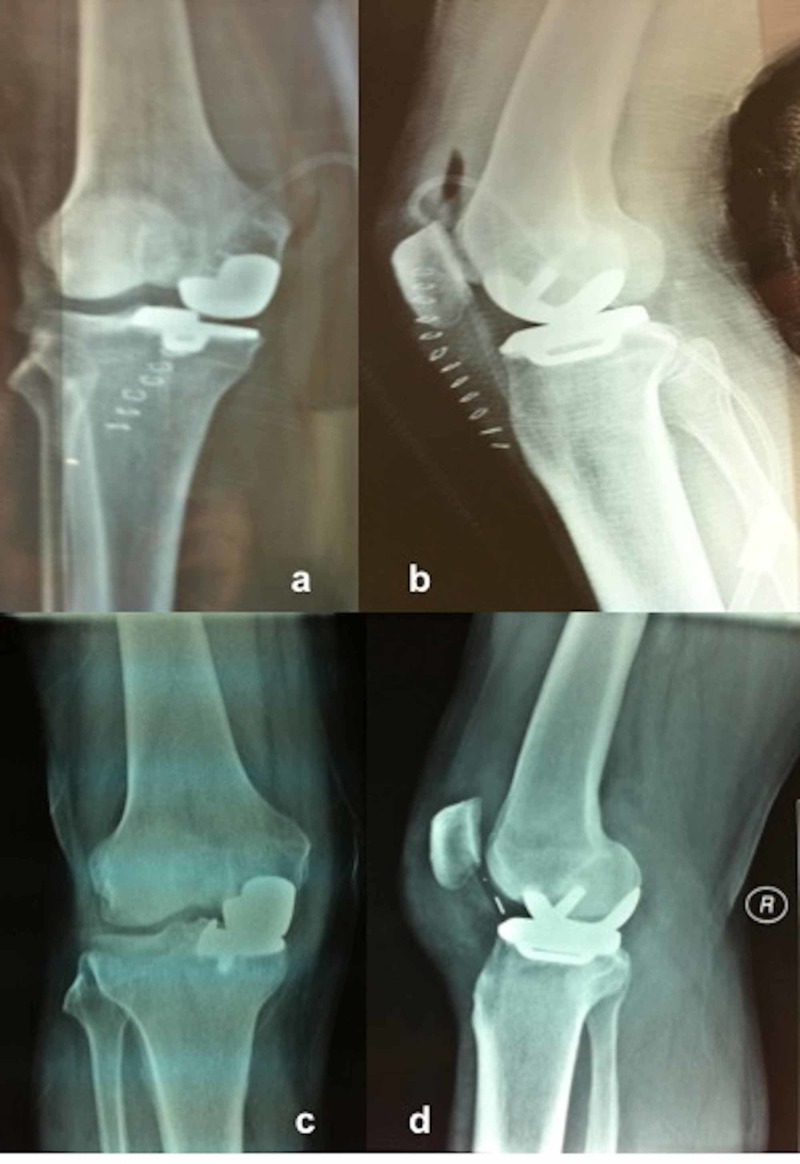
A 60-year-old male patient with UKA due to medial compartment osteoarthritis a,b) Postoperative radiographs of the right knee; c,d) Radiographs of mobile-bearing dislocation at six weeks postoperatively (radiopaque wire of the bearing is seen at the anterior aspect of the knee joint) UKA: unicompartmental knee arthroplasty

**Figure 4:**
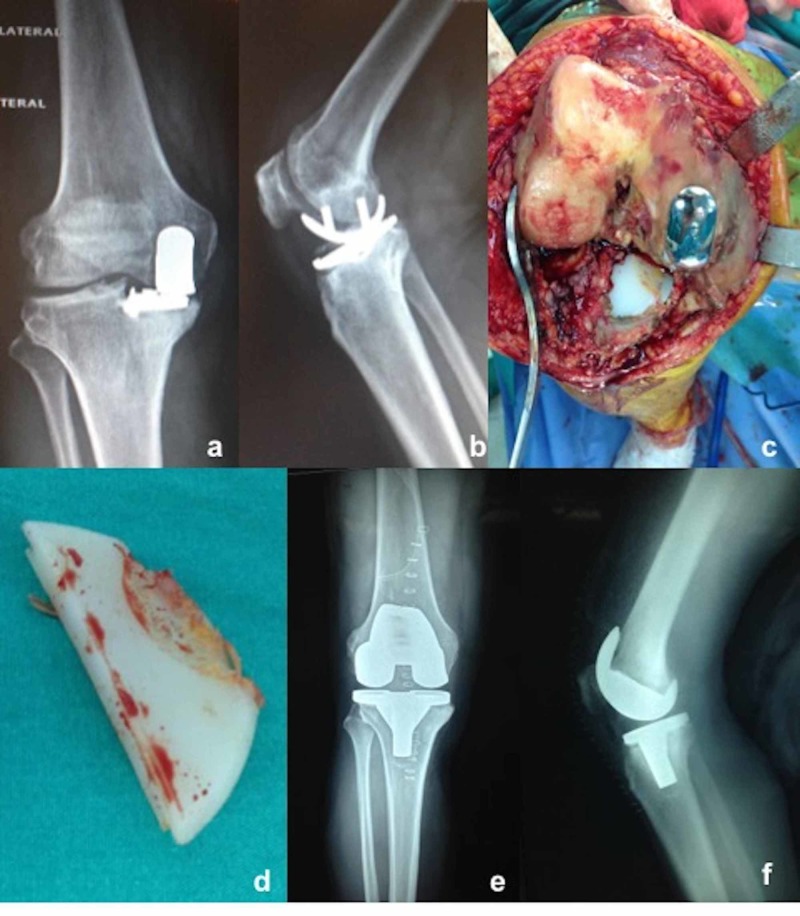
a,b) Tibial and femoral component mismatch (lateralized femoral component and medialized and small tibial component); c) Intraoperative view of the joint; d) Excessive wear on the medial aspect of bearing; e,f) Radiographs after conversion to TKA TKA: total knee arthroplasty

**Figure 5:**
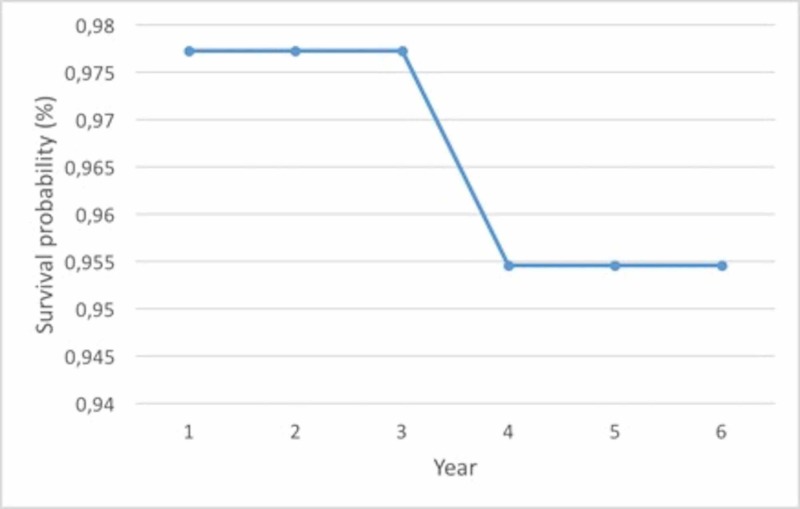
Kaplan-Meier survivorship curve with reoperation (either exchange of bearing or conversion to total knee arthroplasty) as the endpoint. The cumulative survival rate is 95.4% at six years (95% confidence interval: 94.0%-100.0%).

None of the patients experienced any intraoperative complications. During follow-up, one male patient had bearing dislocation at six weeks postoperatively, which was initially managed by closed reduction under sedative anesthesia. He returned to his pre-dislocation level of activity, however, he had insert redislocation after four weeks of closed reduction, which was also managed by anatomical and thicker bearing replacement via revision surgery (Figure 3). In one female patient at four years postoperatively, conversion to TKA due to a tibiofemoral component size mismatch, which caused excessive wear on the medial aspect of the bearing and lateral compartment osteoarthritis, has been performed (Figure 4) (Table 4). Two female patients had persistent anterior and medial knee pain postoperatively without any sign of bearing dislocation or implant-related problems, and complete pain relief was achieved with continuous physiotherapy at one year postoperatively. Though patellofemoral joint arthritis has been suspected in three knees, they are under close observation without any treatment due to the absence of clinical symptoms. None of the patients had deep vein thrombosis, infection, implant loosening, osteolysis, implant-related fracture, or lateral compartment osteoarthritis. On the Kaplan-Meier survival analysis, the median follow-up was 5.9 years and the five-year survival rate of the implants was 95% (Figure 5). Considering the small case number, the result should be interpreted with caution.

**Table 4:** Details of two cases with complications TKA: total knee arthroplasty

Case	Complication	Time to reoperation	Intraoperative Findings	Procedure
1	Bearing dislocation	3 months	MCL laxity	Bearing exchange with thicker one, good results at 6 years
2	Tibial and femoral component mismatch	4 years	Lateral osteoarthritis, Excessive bearing wear	Conversion to TKA

## Discussion

In the present study, we evaluated the mid-term results of mobile-bearing UKA for the treatment of medial compartment osteoarthritis. One bearing dislocation was revised and one conversion to TKA was performed during a 5.9-year follow-up. Overall, our results included a mean five-year survival rate of 95.4%. Clinical outcomes were excellent, with a mean postoperative ROM, OKS, and KSS of 121°, 53, and 90, respectively. This clinical score improvement is consistent in most recent studies with Oxford mobile-bearing UKA implants for medial compartment osteoarthritis.

There is still controversy about the best treatment options for patients with medial compartment-involved knee osteoarthritis. In cases of symptomatic medial compartment knee OA, correcting osteotomies, such as a high tibial osteotomy (HTO) and distal femoral osteotomy, total knee arthroplasties (TKA), or unicompartmental knee arthroplasties (UKA), have been considered the treatment of choice [15-16]. Though TKA was the most commonly performed procedure in operative interventions for degenerative knee joints, there have been higher complication rates as compared to UKA [17]. Moreover, advances in surgical technique and instruments with improvement in clinical outcomes and the long-term survival rate has increased UKA’s popularity and has led to a further increase in the applied frequency of the technique [5,16,18].

The survival rate after UKA depends on many factors. In the current study, the survival rate of the prosthesis was 95.4% at 5.9 years with a mean age of 54 ± 6.1. In similar studies, Tadros et al. reported a 93% survival rate at 4.7 years with an age of older than 57.9 years [19] while Clement et al. reported an 87.7% survival rate at five years with a mean age of 69.5 years [20], and Pandit et al. 97.3% at seven years with a mean age of 66.4 years [21]. In these studies, younger age (<60 years) was accepted as a predictor of failure, and they explain the reason as younger people and males expect greater improvement in knee function than the prosthesis can offer. However, there is no consensus on the application of the procedure in young and active patients. Pandit et al. suggested that age should not be a contraindication for UKA surgery [22]. Furthermore, in this study, a 95.4% survival rate at 5.9 years, regardless of age, has disproved those scientific publications.

On the other hand, it is discouraging to note that a high activity level increases the risk of developing complications, which usually require a reoperation. Bearing dislocation is the most common cause of failure that requires reoperation after UKA and frequently occurs in patients who have a high level of activity [23]. The possible causes of a bearing dislocation include inappropriate gap balancing between flexion and extension, implant malpositioning, excessive release of the medial collateral ligament, and posterior impingement of bearing by the remaining meniscus or osteophytes [24]. Dahl et al. reported a three times higher reoperation rate in patients <55 years [25], and Kuipers et al. reported a 2.2 times higher revision risk in patients >69 years [26]. In the current study, a bearing dislocation occurred in a male patient. Probably the reason for our complication is the use of early-designed non-anatomical mobile-bearing implants in a patient with a high activity level. Because in terms of higher incidence of dislocation, the Oxford group developed a new anatomical bearing, which had an extended length of a medial wall to protect further dislocations. Furthermore, the new bearing increased the amount of rotation that the bearing had to undergo and the anteromedial corner of the bearing has been reduced to decrease the anterior overhang in the extension. Moreover, the majority of the study population in the current study consists of the female gender, and most Turkish women have a standard lifestyle where high flexion of the knee is not essential as compared to Asian and Western counterparts. Accordingly, mobile-bearing UKA may be a proper solution for osteoarthritis in Turkish women.

In addition, the experience of the surgeon could provide a significant contribution to achieving success. Because the level of experience of the surgeon is considered a key factor in the overall survival of the implants and satisfactory outcomes in many scientific reports. In supporting studies, it is reported that low-volume UKA performing centers have caused higher revision rates [20,27]. Bini et al. evaluated the surgeon volume effects on revision rates and declared a yearly volume of less than 12 UKA is a significant risk for failure [28]. In a high-volume study reported by Baker et al., 23400 UKA were evaluated and concluded that high-volume centers and surgeons specialized in UKA showed superior outcomes, and the minimum number of UKA’s per surgeon should be more than 13 per year [29]. In the current study, the reason for conversion to TKA in one patient is considered as a tibial and femoral component size mismatch. In our practice, 11 UKA per year is not as low as the volume suggested in the aforementioned studies, however, there may be mechanical, technical, and surgeon-related problems in terms of component size and placement. So these results underline the existence of a learning curve for UKA and that minimally invasive Oxford Phase 3 UKA is a demanding procedure that nonetheless has the potential to achieve satisfying surgical results.

Nonetheless, there are some limitations to our study. First, we retrospectively evaluated the patients, which brings the possibility of selection bias and there was no control group, thus limiting the strength of the current analysis. Second, the study lacked a comparison with fixed-bearing UKA. Third, three of 37 patients included in this study were male, so we could not compare the gender demographics and clinical and radiological effects on survivorship. Lustig et al. reported that the gender effect on the outcome of UKA has no difference in terms of the range of motion, radiologic progression of arthritis, and alignment [30]. There is no comprehensive study about gender effect on the results of UKA; there may be further investigations required on this subject. Fourth, this cohort was a set of consecutive patient series from a single surgeon in the second decade of his practice and a highly specific patient group. Fifth, the study population was also small due to the strict selection criteria. A larger sample size might be better for finding the prevalence of complications related to mobile-bearing UKA. And last, the mean follow-up period of this study was six years, which may be relatively short for a primary arthroplasty series; therefore, further studies are needed to elucidate the long-term outcomes of this technique.

## Conclusions

In conclusion, our study results suggest that UKA can yield promising mid-term results in terms of the mobile-bearing type and regardless of age if it remains faithful to the surgical technique. However, we recommend large-scale and long-term, prospective, clinical studies to confirm the efficacy and safety of this technique.

## References

[1] McKeever DC (1960). Tibial plateau prosthesis. Clin Orthop.

[2] Marmor L (1979). Marmor modular knee in unicompartmental disease: minimum four-year follow-up. J Bone Joint Surg Am.

[3] Marmor L (1988). Unicompartmental arthroplasty of the knee with a minimum ten-year follow-up period. Clin Orthop.

[4] Murray DW, Goodfellow JW, O'Connor JJ (1998). The Oxford medial unicompartmental arthroplasty: a ten-year survival study. J Bone Joint Surg Br.

[5] Alnachoukati OK, Barrington JW, Berend KR, Kolczun MC, Emerson RH, Lombardi AV Jr, Mauerhan DR (2018). Eight hundred twenty-five medial mobile-bearing unicompartmental knee arthroplasties: the first 10-year US multi-center survival analysis. J Arthroplasty.

[6] Panzram B, Bertlich I, Reiner T, Walker T, Hagmann S, Gotterbarm T (2017). Cementless Oxford medial unicompartmental knee replacement: an independent series with a 5-year-follow-up. Arch Orthop Trauma Surg.

[7] Khanna G, Levy BA (2007). Oxford unicompartmental knee replacement: literature review. Orthopedics.

[8] Price AJ, Svard U (2011). A second decade lifetable survival analysis of the Oxford unicompartmental knee arthroplasty. Clin Orthop Relat Res.

[9] Svärd UC, Price AJ (2001). Oxford medial unicompartmental knee arthroplasty. A survival analysis of an independent series. J Bone Joint Surg Br.

[10] Ashraf T, Newman JH, Desai VV, Beard D, Nevelos JE (2004). Polyethylene wear in a non-congruous unicompartmental knee replacement: a retrieval analysis. Knee.

[11] Parratte S, Argenson JN, Pearce O, Pauly V, Auquier P, Aubaniac JM (2009). Medial unicompartmental knee replacement in the under-50s. J Bone Joint Surg Br.

[12] Argenson JN, Parratte S (2006). The unicompartmental knee: design and technical considerations in minimizing wear. Clin Orthop Relat Res.

[13] Dawson J, Fitzpatrick R, Carr A, Murray D (1996). Questionnaire on the perceptions of patients about total hip replacement. J Bone Joint Surg Br.

[14] Insall JN, Dorr LD, Scott RD, Scott WN (1989). Rationale of the knee society clinical rating system. Clin Orthop Relat Res.

[15] Lyons MC, MacDonald SJ, Somerville LE, Naudie DD, McCalden RW (2012). Unicompartmental versus total knee arthroplasty database analysis: is there a winner?. Clin Orthop Relat Res.

[16] Arirachakaran A, Choowit P, Putananon C, Muangsiri S, Kongtharvonskul J (2015). Is unicompartmental knee arthroplasty (UKA) superior to total knee arthroplasty (TKA)? A systematic review and meta-analysis of randomized controlled trial. Eur J Orthop Surg Traumatol.

[17] Brown NM, Sheth NP, Davis K, Berend ME, Lombardi AV, Berend KR, Della Valle CJ (2012). Total knee arthroplasty has higher postoperative morbidity than unicompartmental knee arthroplasty: a multicenter analysis. J Arthroplasty.

[18] Koh IJ, Kim MW, Kim JH, Han SY, In Y (2015). Trends in high tibial osteotomy and knee arthroplasty utilizations and demographics in Korea from 2009 to 2013. J Arthroplasty.

[19] Tadros BJ, Dabis J, Twyman R (2018). Short-term outcome of unicompartmental knee arthroplasty in the octogenarian population. Knee Surg Sports Traumatol Arthrosc.

[20] Clement ND, Duckworth AD, MacKenzie SP, Nie YX, Tiemessen CH (2012). Medium-term results of Oxford phase-3 medial unicompartmental knee arthroplasty. J Orthop Surg (Hong Kong).

[21] Pandit H, Jenkins C, Barker K, Dodd CA, Murray DW (2006). The Oxford medial unicompartmental knee replacement using a minimally-invasive approach. J Bone Joint Surg Br.

[22] Pandit H, Jenkins C, Gill HS, Smith G, Price AJ, Dodd CA, Murray DW (2011). Unnecessary contraindications for mobile-bearing unicompartmental knee replacement. J Bone Joint Surg Br.

[23] Kim SJ, Postigo R, Koo S, Kim JH (2014). Causes of revision following Oxford phase 3 unicompartmental knee arthroplasty. Knee Surg Sports Traumatol Arthrosc.

[24] Lewold S, Goodman S, Knutson K, Robertsson O, Lidgren L (1995). Oxford meniscal bearing knee versus the Marmor knee in unicompartmental arthroplasty for arthrosis. A Swedish multicenter survival study. J Arthroplasty.

[25] W-Dahl A, Robertsson O, Lidgren L, Miller L, Davidson D, Graves S (2010). Unicompartmental knee arthroplasty in patients aged less than 65. Acta Orthop.

[26] Kuipers BM, Kollen BJ, Bots PC, Burger BJ, van Raay JJ, Tulp NJ, Verheyen CC (2010). Factors associated with reduced early survival in the Oxford phase III medial unicompartment knee replacement. Knee.

[27] Bell SW, Stoddard J, Bennett C, London NJ (2014). Accuracy and early outcomes in medial unicompartmental knee arthroplasty performed using patient specific instrumentation. Knee.

[28] Bini S, Khatod M, Cafri G, Chen Y, Paxton EW (2013). Surgeon, implant, and patient variables may explain variability in early revision rates reported for unicompartmental arthroplasty. J Bone Joint Surg Am.

[29] Baker P, Jameson S, Critchley R, Reed M, Gregg P, Deehan D (2013). Center and surgeon volume influence the revision rate following unicondylar knee replacement: an analysis of 23,400 medial cemented unicondylar knee replacements. J Bone Joint Surg Am.

[30] Lustig S, Barba N, Magnussen RA, Servien E, Demey G, Neyret P (2012). The effect of gender on outcome of unicompartmental knee arthroplasty. Knee.

